# Analysis of clinical features and early warning signs in patients with severe COVID-19: A retrospective cohort study

**DOI:** 10.1371/journal.pone.0235459

**Published:** 2020-06-26

**Authors:** Xinkui Liu, Xinpei Yue, Furong Liu, Le Wei, Yuntian Chu, Honghong Bao, Yichao Dong, Wenjie Cheng, Linpeng Yang

**Affiliations:** 1 Medical Records Management Department, The First Affiliated Hospital of Zhenghzou University, Zhengzhou, Henan, China; 2 School of Health Care Management, Tongji Medical University, Huazhong University of Science and Technology, Wuhan, Hubei, China; Erasmus Medical Center, NETHERLANDS

## Abstract

Coronavirus disease 2019 (COVID-19) was first identified in Wuhan, China, in December 2019. Although previous studies have described the clinical aspects of COVID-19, few studies have focused on the early detection of severe COVID-19. Therefore, this study aimed to identify the predictors of severe COVID-19 and to compare clinical features between patients with severe COVID-19 and those with less severe COVID-19. Patients admitted to designated hospital in the Henan Province of China who were either discharged or died prior to February 15, 2020 were enrolled retrospectively. Additionally, patients who underwent at least one of the following treatments were assigned to the severe group: continuous renal replacement therapy, high-flow oxygen absorption, noninvasive and invasive mechanical ventilation, or extracorporeal membrane oxygenation. The remaining patients were assigned to the non-severe group. Demographic information, initial symptoms, and first visit examination results were collected from the electronic medical records and compared between the groups. Multivariate logistic regression analysis was performed to determine the predictors of severe COVID-19. A receiver operating characteristic curve was used to identify a threshold for each predictor. Altogether,104 patients were enrolled in our study with 30 and 74 patients in the severe and non-severe groups, respectively. Multivariate logistic analysis indicated that patients aged ≥63 years (odds ratio = 41.0; 95% CI: 2.8, 592.4), with an absolute lymphocyte value of ≤1.02×10^9^/L (odds ratio = 6.1; 95% CI = 1.5, 25.2) and a C-reactive protein level of ≥65.08mg/L (odds ratio = 8.9; 95% CI = 1.0, 74.2) were at a higher risk of severe illness. Thus, our results could be helpful in the early detection of patients at risk for severe illness, enabling the implementation of effective interventions and likely lowering the morbidity of COVID-19 patients.

## Introduction

In December 2019, a local cluster of patients with pneumonia of unknown etiology was observed; subsequently, similar cases were observed nationally. On January 7, 2020, the virus strain causing pneumonia was successfully isolated by researchers [1,2]. This virus is genetically related to the virus responsible for the severe acute respiratory syndrome-related coronavirus (SARS-CoV) outbreak in 2003 [3]; consequently, the new virus was named the severe acute respiratory syndrome coronavirus 2 (SARS-CoV-2) by the International Committee on Taxonomy of Viruses on February 11. On the same day, the World Health Organization (WHO) proclaimed the official name of the disease caused by the new virus as coronavirus disease (COVID-19) [4].

As COVID-19 continued to spread and developed into an epidemic, the WHO declared COVID-19 a “public health emergency of international concern” [5]. As of March 9, there were 80,754 confirmed cases reported in China [6], of which 1,272 cases were in the Henan province [7]. A study by Zhou et al. reported the risk factors for mortality of COVID-19 inpatients in Wuhan [8], but the thresholds for each risk factor was not given. To our knowledge, there have been no studies to predict patients with severe COVID-19 from the results of their first visit.

The objective of this study was to determine the risk factors for severe COVID-19 based on the test results from the patient’s first visit, and describe the clinical features in patients with severe COVID-19.

## Materials and methods

### Study population

Our study population consisted of confirmed COVID-19 patients who were either discharged or died prior to February 15, 2020. Our study was launched on March 1, 2020. Participants with the following missing information were excluded from the study: age, date of first symptom, contact date, or examination data. All demographic and clinical information was collected retrospectively from the electronic medical records (EMR).

Patients who underwent at least one of the following procedures were assigned to the severe group: continuous renal replacement therapy (CRRT), high flow oxygen absorption, noninvasive and invasive mechanical ventilation, and extracorporeal membrane oxygenation (ECMO). The remaining participants were assigned to the non-severe group.

### Data collection

Data obtained from the EMR included age, sex, contact history, latest contact date, primary symptoms and date of occurrence, comorbidities, and the results of routine blood tests as well as liver function, kidney function, C-reactive protein (CRP), and procalcitonin tests from the initial visit. Confidentiality of information was maintained by removing personal identifiable information. The medical research ethics committee of The First Affiliated Hospital of Zhenghzou University approved this study.

The following comorbidities were noted among our participants: respiratory diseases such as chronic obstructive pulmonary disease (COPD), asthma, and interstitial pneumonia; metabolic diseases such as diabetes mellitus; cardiovascular disease (CVD) such as hypertension and coronary heart disease; and neurological diseases such as cerebral hemorrhage and infarction.

A clustered onset was defined as two or more cases with fever or respiratory symptoms in a confined area, such as a home, office, school class, or similar setting, within the previous two weeks. The incubation period was defined as the time between the date of occurrence of primary symptoms and latest contact date with known infected individuals.

Data from the EMR were entered using EpiData and confirmed twice. Every case was entered independently by two different data entry clerks and consistency was verified by a supervisor. Three members of our research group were responsible for reviewing each data entry for which conflicting data had been entered. The doi link is dx.doi.org/10.17504/protocols.io.bfpejmje.

### Statistical analysis

All data were entered and managed in EpiData. SPSS 19.0 was utilized for cleaning and analyzing the data. As the quantitative data did not follow a normal distribution, the data were expressed as medians and interquartile ranges (IQRs) after applying the Mann-Whitney U test for comparison between the groups. Qualitative data were expressed using counts and percentages and compared using the chi-squared or Fisher’s exact test. Thresholds were calculated using receiver operating characteristic (ROC) curves. Independent effects were shown using multivariate logistic regression. Based on the univariate analysis results and clinical experience, five variables were chosen for the multivariable analysis including age, comorbidity, lymphocyte count, CRP levels, and procalcitonin levels. The significance level was set at 0.05.

## Results

In the Henan province, there were a total of 410 confirmed COVID-19 patients with a final outcome of discharge or death prior to February 15. After excluding cases with missing values, 74 participants were assigned to then on-severe group and 30 participants were assigned to the severe group.

The median age of the study participants was 42.0(IQR = 31.0–55.0) years, ranging from 5 to 85 years. There were 63 male patients, accounting for 60.6% of all participants and 41 female patients, accounting for 39.4% of all participants. Of the 104 participants, 84 (80.8%) had a history of traveling or residing in either Wuhan or the surrounding areas, or other communities with reported COVID-19 cases, while 38 participants (36.5%) had contact with individuals who were experiencing fever or respiratory symptoms and had recently been to Wuhan or its surrounding areas, or other communities with reported cases. Furthermore, 17 (16.3%) patients had a clustered onset, 24 (23.1%) had a history of contact with a COVID-19 patient, and 6 (5.8%) had an unknown history of exposure. The median incubation period for the entire group was 5.0 (IQR = 3.0–9.0) days, ranging from1 days to14 day. An incubation period of less than 7 days was observed in 70.5% of the patients. Further, 29 (27.9%) participants had underlying diseases, with cardiovascular disease being the most common underlying disease, followed by metabolic and respiratory disease. The main symptoms of the participants included fever, cough, and fatigue. Fever was the most common initial symptom, manifesting in 79 patients (76.0%) on admission (Table 1).

**Table 1:** Baseline demographics and epidemiological and clinical status of participants.

Basic information	Statistics			*P*
	All patients	Non-severe group	Severe group	
Age, median (IQR)	42.0 (31.0–55.0)	40.0 (32.0–55.0)	55.0 (31.0–72.0)	0.005
Sex				0.509
Male, n (%)	63(60.6)	43 (58.1)	20 (66.7)	-
Female, n (%)	41(39.4)	31 (41.9)	10 (33.3)	-
Exposure history				
Travelling in Wuhan, n (%)	84 (80.8)	65 (87.8)	19 (63.3)	0.006
Contact with patient in Wuhan, n (%)	38 (36.5)	31 (41.9)	7 (23.3)	0.115
Clustered onset, n (%)	17 (16.3)	13 (17.6)	4 (13.3)	0.772
Contact with COVID-19, n (%)	24 (23.1)	17 (23.0)	7 (23.3)	1.000
Unknown exposure, n (%)	6 (5.8)	0 (0.0)	6 (20%)	<0.001
Incubation period, n (min-max)	5.0 (3.0–9.0)	5.0 (2.0–9.0)	5.0 (3.0–8.0)	0.956
Comorbidity, n (%)	29 (27.9)	16 (21.6)	13 (43.3)	0.032
Respiratory disease, n (%)	10 (9.6)	6 (8.1)	4 (13.3)	0.426
Metabolic disease, n (%)	12 (11.5)	6 (8.1)	6 (20.0)	0.099
Cardiovascular disease, n (%)	23 (22.1)	12 (16.2)	11 (36.7)	0.036
Neurological disease, n (%)	1 (1.0)	1 (1.4)	0 (0.0)	1.000
Others, n (%)	2 (1.9)	1 (1.4)	1 (3.3)	0.496
Symptoms				
Fever, n (%)	96 (92.3)	66 (89.2)	30 (100)	0.065
Fatigue, n (%)	50 (48.1)	37 (50.0)	13 (43.3)	0.665
Cough, n (%)	66 (63.5)	45 (60.8)	21 (70.0)	0.501
Expectoration, n (%)	33 (31.7)	24 (32.4)	9 (30.0)	0.822
Asthma, n (%)	12 (11.5)	6 (8.1)	6 (20.0)	0.099
Nasal obstruction, n (%)	7 (6.7)	4 (5.4)	3 (10.0)	0.670
Runny nose, n (%)	9 (8.7)	6 (8.1)	3 (10.0)	1.000
Pharyngalgia, n (%)	15(14.4)	13 (17.6)	2(6.7)	0.221
Chest distress, n (%)	22 (21.2)	13 (17.6)	9 (30.0)	0.189
Headache, n (%)	11 (10.6)	8 (10.8)	3(10.0)	1.000
Dizziness, n (%)	9 (8.7)	7 (9.5)	2 (6.7)	0.726
Poor appetite, n (%)	13 (12.5)	9 (2.2)	4 (13.3)	1.000
Diarrhea, n (%)	7 (6.7)	5 (6.8)	2 (6.7)	1.000
Muscle and joint pain, n (%)	15 (14.4)	9 (12.2)	6 (20.0)	0.359
Dyspnea, n (%)	9 (8.7)	2 (2.7)	7 (23.3)	0.001

^a^Quantitative data are expressed as median and interquartile range (IQR); qualitative data are expressed as count and percentages.

The median age in the non-severe group (55 years) was significantly higher than in the severe group (40 years). Underlying diseases manifested in 13 (43.3%) patients in the severe group, which was higher than in the non-severe group, and the difference was statistically significant (P<0.05). The proportion of patients with cardiovascular disease was also greater in the severe group than in the non-severe group, and the difference was statistically significant (P <0.05). With regard to symptoms, the proportion of patients with dyspnea was significantly higher in the severe group than in the non-severe group (P <0.05). The proportions of patients with fever, cough, asthma, and chest distress were greater in the severe group; however, these differences were not significant. Other symptoms demonstrated no quantifiable difference between the severe and non-severe groups (Table 1).

The majority of factors examined at first admission indicated a non-significant difference between the two groups; however, direct bilirubin, lactate dehydrogenase, CRP, and procalcitonin levels were significantly higher in the severe group than in the non-severe group (P <0.05). Additionally, the absolute value of lymphocyte count was also significantly lower in the severe group than in the non-severe group (P <0.05) as shown in Table 2.

**Table 2:** Initial laboratory results of COVID-19 patients.

Index	Median (IQR)			P
	All Patients	Non-severe Group	Severe Group	
Blood routine testing				
Leukocyte count, 10^9/L	4.78 (3.37–6.30)	4.78 (3.35–6.17)	4.55 (3.78–7.58)	0.299
RBC count, 10^12/L	4.50 (4.08–4.91)	4.51 (4.86–4.08)	4.47 (4.06–5.00)	0.553
Platelet count, 10^9/L	172.50 (139.00–206.00)	174.50 (141.25–206.00)	156.50 (130.75–207.25)	0.581
Lymphocyte count, 10^9/L	1.12 (0.80–1.45)	1.29 (0.97–1.57)	0.91 (0.59–1.11)	0.002
Neutrophil count, 10^9/L	3.05 (1.93–4.76)	3.01 (1.94–4.49)	3.97 (1.90–6.29)	0.213
Eosinophils count,10^9/L	0.01 (0.00–0.03)	0.01 (0.00–0.03)	0.01 (0.00–0.03)	0.345
Monocyte count, 10^9/L	0.32 (0.20–0.48)	0.32 (0.20–0.48)	0.28 (0.19–0.49)	0.836
Lymphocyte, %	23.15 (10.54–33.65)	24.55 (15.00–35.65)	20.15 (7.53–32.83)	0.368
Neutrophil, %	65.10 (54.75–77.05)	65.30 (54.90–73.50)	62.96 (54.40–86.65)	0.231
Eosinophils, %	0.20 (0.00–0.40)	0.20 (0.00–0.40)	0.10 (0.00–0.43)	0.226
Monocytes, %	6.60 (4.13–9.53)	6.50 (4.30–9.00)	7.00 (4.00–10.00)	0.897
Liver function				
Alanine aminotransferase, U/L	22.00 (15.00–40.00)	21.00 (15.00–40.00)	27.90 (17.00–53.58)	0.212
Aspartate aminotransferase, U/L	26.25 (20.23–35.00)	25.00 (20.00–33.00)	30.00 (23.00–39.00)	0.120
Total bilirubin, μmol/L	10.44 (6.60–14.90)	10.44 (7.01–14.30)	10.45 (5.80–17.27)	0.758
Direct bilirubin,μmol/L	3.30 (2.40–4.80)	3.20 (1.95–4.40)	4.27 (3.03–5.78)	0.015
Indirect bilirubin,μmol/L	7.06 (3.93–10.18)	7.02 (4.35–10.45)	7.40 (2.80–10.20)	0.495
Lactate dehydrogenase,U/L	203.00 (173.00–266.00)	198.00 (172.00–226.00)	249.59 (188.75–403.00)	0.004
Creatine kinase, U/L	75.00 (53.00–144.00)	77.00 (52.50–121.79)	74.00 (55.00–235.00)	0.286
Albumin,g/L	40.60 (36.80–44.40)	41.40 (37.30–44.90)	39.90 (34.70–43.80)	0.098
Kidney function				
Creatinine, μmol/L	62.80 (52.84–71.00)	60.50 (52.20–71.00)	66.90 (52.97–74.00)	0.383
Urea, mmol/L	4.20 (3.25–6.00)	4.20 (3.22–5.30)	4.30 (3.35–8.46)	0.483
C-reactive protein,mg/L	13.62 (5.26–31.37)	10.61 (4.08–18.78)	31.22 (11.63–92.52)	0.001
Procalcitonin,μg/L	0.07 (0.04–0.19)	0.05 (0.03–0.09)	0.17 (0.06–0.21)	0.001

**Fig 1:**
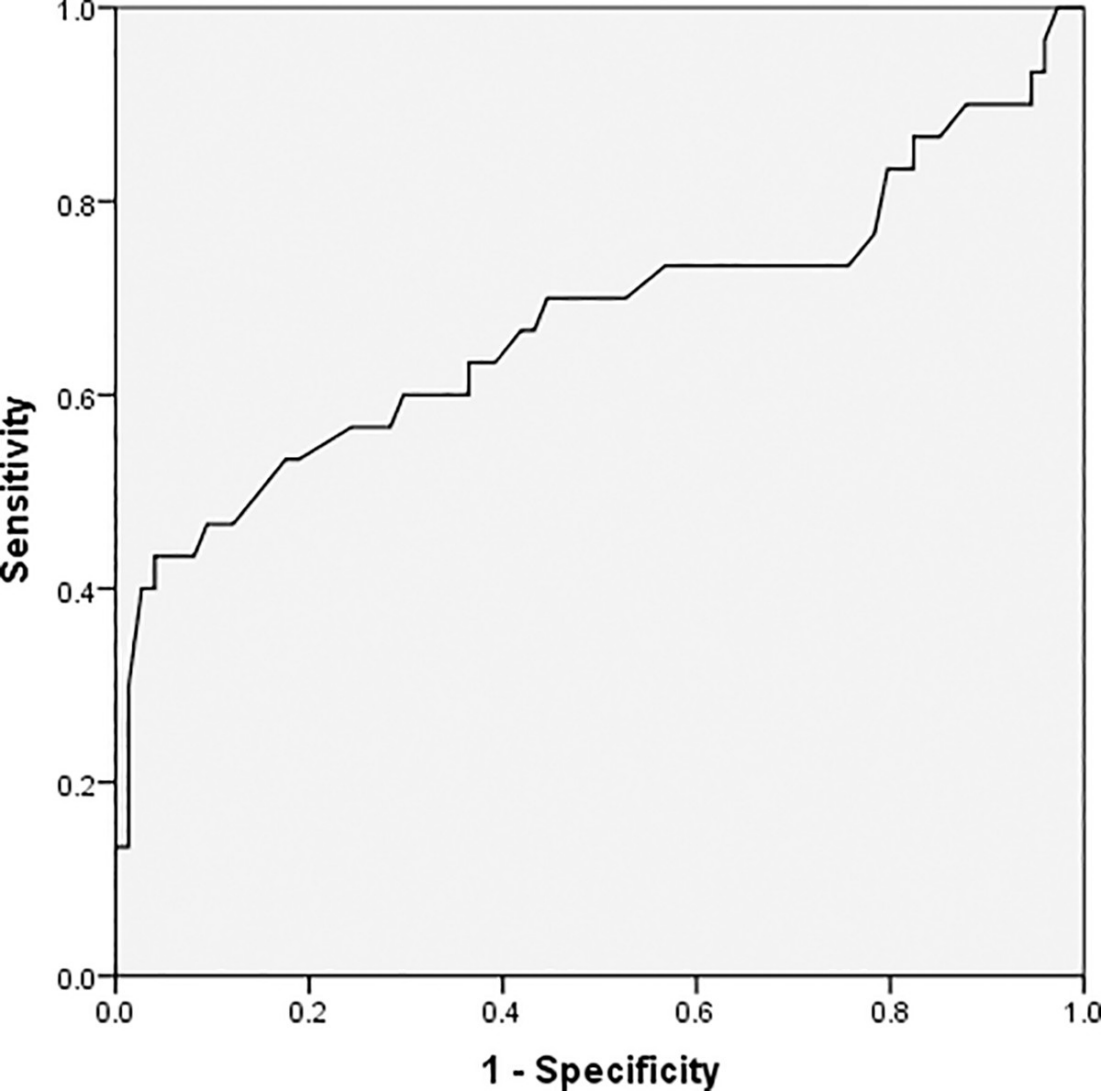
Receiver operating characteristic curve for age.

**Fig 2:**
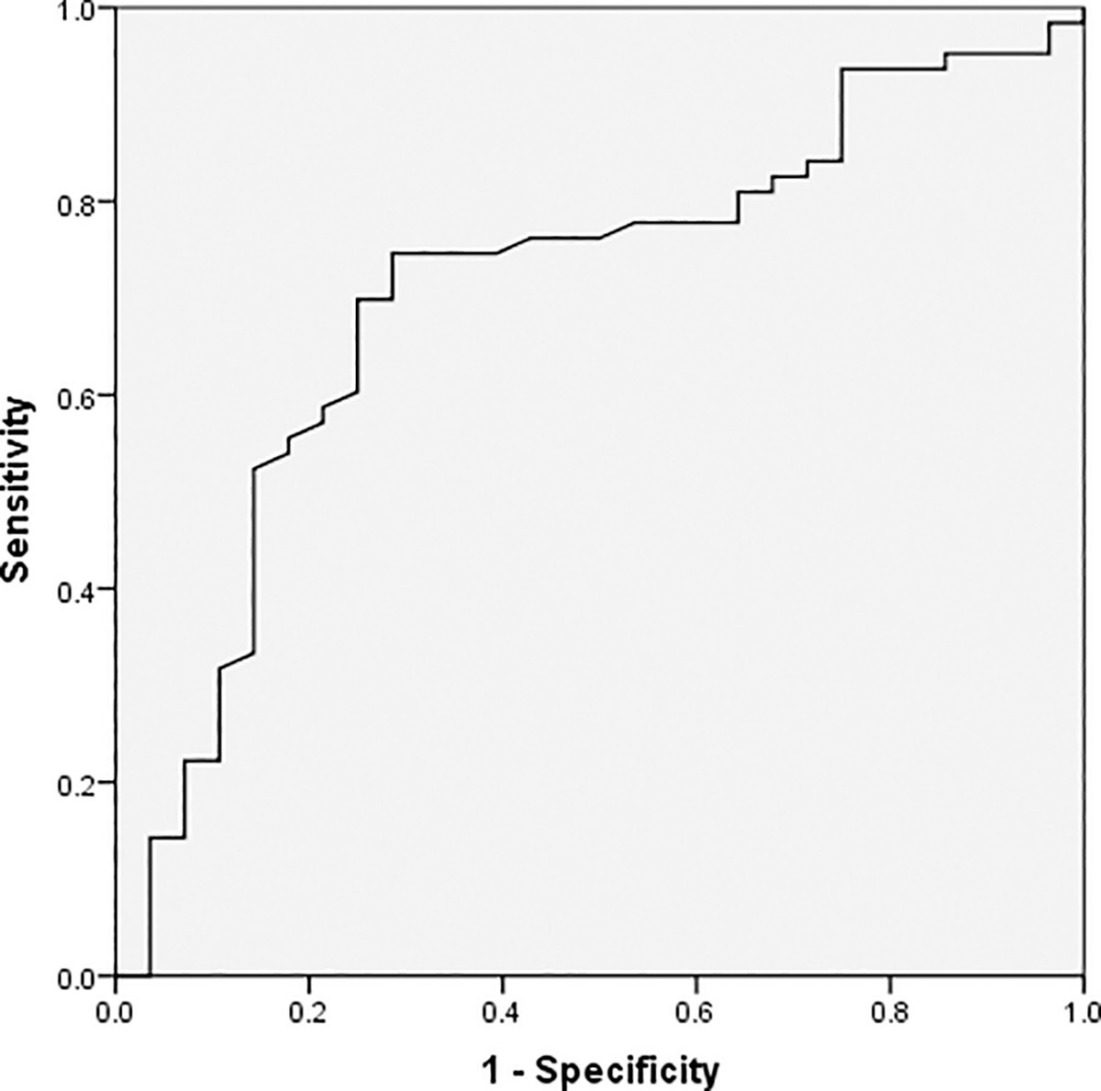
Receiver operating characteristic curve for absolute lymphocyte value.

**Fig 3:**
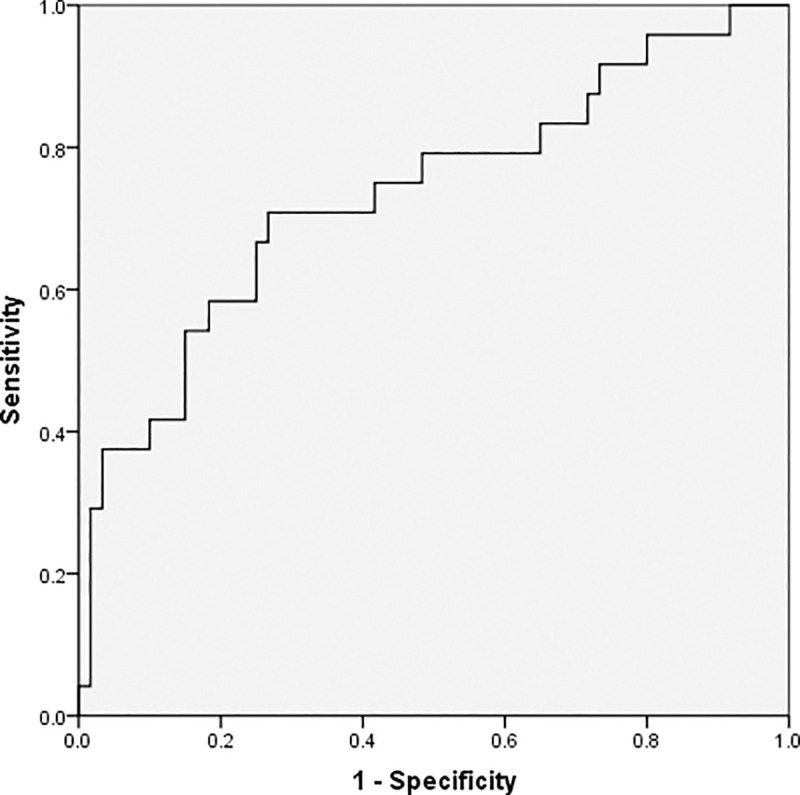
Receiver operating characteristic curve for C-reactive protein levels.

**Fig 4:**
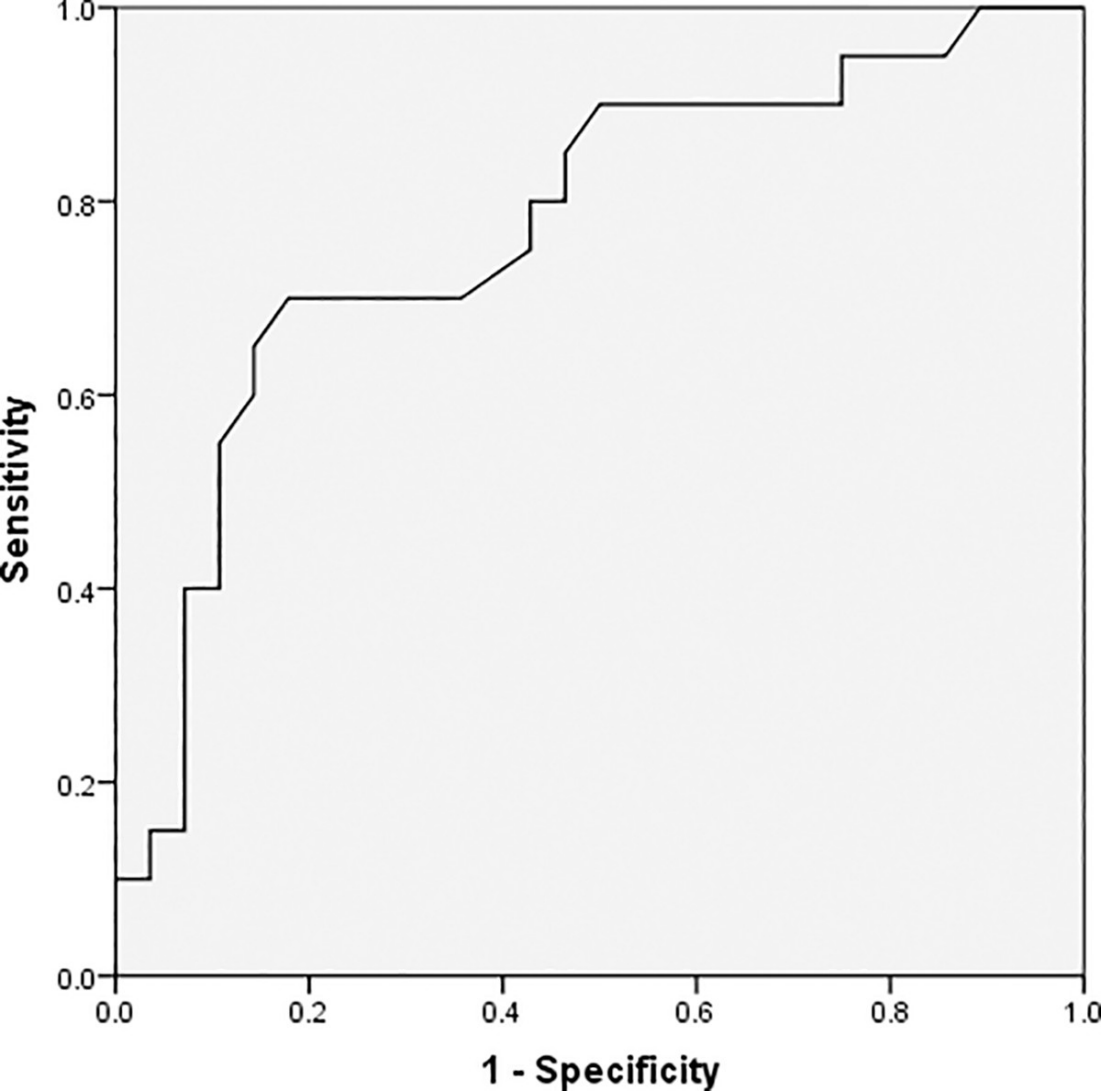
Receiver operating characteristic curve for procalcitonin levels.

As demonstrated by the ROC curves using a single predictor, age, the absolute lymphocyte value, CRP, and procalcitonin are valuable predictors for detecting severe conditions in patients; the area under the curve (AUC) for these predictors were 0.676, 0.708, 0.734, and 0.773, respectively. Further, the thresholds for these factors were 63 for age, 1.02×10^9/L for lymphocyte, 65.08mg/L for CRP, and 0.12μg/L for procalcitonin (Figs 1–4 and Table 3).

**Table 3:** Analysis of the receiver operating characteristic curve for a single predictor.

Variables	Threshold	AUC(95%CI)	Youden index	*P*
Age, years	63	0.676 (0.542,0.809)	0.393	0.005
Absolute lymphocyte value, 10^9/L	1.02	0.708 (0.592,0.825)	0.460	0.002
C-reactive protein, mg/L	65.08	0.734 (0.607,0.861)	0.492	0.001
Procalcitonin, μg/L	0.12	0.773 (0.634,0.912)	0.567	0.001

^a^AUC, area under the receiver operating characteristic curve; CI, confidence interval.

**Fig 5:**
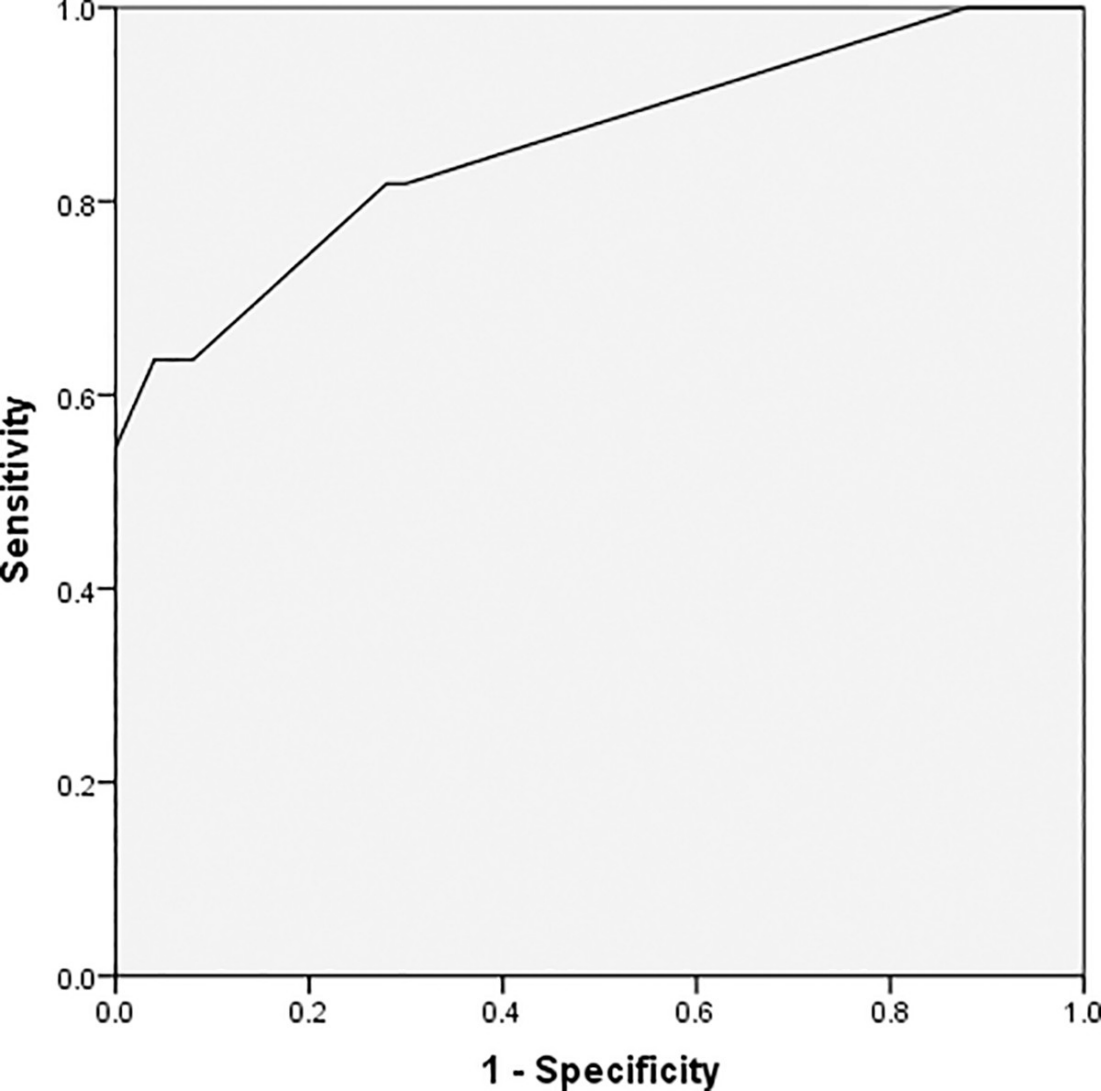
Receiver operating characteristic curve of the multivariate prediction model.

The results of the multivariate logistic regression indicate that age, lymphocyte, and CRP are independent predictors for an increased risk of severe COVID-19. Patients who are at least 63 years old, with an absolute lymphocyte value less than 1.02×10^9/L, and a CRP greater than 65.08 mg/L are at greater risk of developing severe COVID-19 with corresponding odds ratios of 41.0, 6.1, and 8.9, respectively (Table 4). An analysis of the ROC curves with multiple predictors is shown in Fig 5. These results have a high degree of accuracy as implied by an AUC of 0.858 (95%CI = 0.754, 0.962).

**Table 4:** Multivariate analysis of COVID-19 severity.

Variables	β	SE	OR(95%CI)	*P*
Age≥63 years old	3.714	1.362	41.036(2.843~592.356)	0.006
Absolute lymphocyte value≤1.02×10^9/L	1.806	0.725	6.089(1.469~25.230)	0.013
C-reactive protein≥65.08mg/L	2.183	1.084	8.876(1.016~74.228)	0.044

^a^SE, standard error; OR, odds ratio; CI, confidence interval. Variables were categorized by threshold values, whereas functions were inferred by ranges, not formulas. The effect in this multivariate regression was that the variables were independent from each other.

## Discussion

Our study indicates that age, the absolute lymphocyte count at initial visit, and CRP may be used as predictors during the early stage of diagnosis in patients who are at risk of developing severe COVID-19. In many severe cases, patients have CVD as a comorbidity, dyspnea, and higher concentrations of direct bilirubin, lactate dehydrogenase, and procalcitonin.

In our study, the median age of the 104 participants was 42.0 years (IQR = 31.0–55.0) ranging from 5 to 85 years of age. In addition, for 70.5% of the patient, the incubation period was less than seven days, which indicates that COVID-19 has a short incubation period and may impact both children and adults. The same result shave been reported in previous studies [9]. The median age in the severe group was 55.0 years (IQR = 31.0–72.0), which is higher than the median age in the non-severe group, (median = 40.0; IQR = 32.0–55.0). The threshold for age was 63 years, as indicated in the ROC curve analysis. The threshold age is a predictor for an increased risk of severe COVID-19. In addition, our multivariate logistic regression analysis implied that patients who are older than 63 years are at higher risk of developing severe COVID-19 (OR: 41.0). The trends indicated in our study are similar to those indicated in previous studies [10]. Moreover, studies show that immune responses in older adults are slower, less coordinated, and less efficient, rendering older adults more susceptible to emerging infections [11]. A study by Shahid et al. indicates that the probability of having multiple comorbidities increased the risk of mortality from SARS-CoV-2 in older adults [12].

Our data indicate that 27.9% of patients had an underlying disease, especially cardiovascular and metabolic disorders. The percentage of patients suffering from CVD was higher in the severe group than in the non-severe group. These results have been verified by epidemic reports from the Chinese Center for Disease Control and Prevention [13]. As in previous studies, the principal symptoms of COVID-19 are respiratory symptoms. Some patients also suffer significant cardiovascular damage from COVID-19. Coupled with underlying CVD, these complications may increase the risk of death for some patients. COVID-19 results from a spike protein in the virus binding with the angiotensin-converting enzyme 2 (ACE2) which is highly expressed in the heart and lung tissue. A contributing factor to severe COVID-19 in patient with CVD may be the increased concentration of ACE2 in these patients. As the Middle East respiratory syndrome-related coronavirus (MERS-CoV) and severe acute respiratory syndrome coronavirus (SARS-CoV) may result in acute myocarditis and heart failure, acute coronary syndrome patients infected with the novel coronavirus are at an increased risk of cardiac insufficiency that might lead to severe COVID-19 or death [14].

The most common symptoms of COVID-19 in our study were fever (92.3%), cough (63.5%), and fatigue (48.1%). Some patients also experienced dizziness (8.7%), a runny nose (8.7%), nasal obstruction (6.7%), and diarrhea (6.7%). These numbers are similar to those described in previous studies [15,16]. Our study indicates that in 76.0% of the patients, the initial symptom was fever. In addition, 92.3% of the patients had fever during the inpatient period; of those, all patients with severe COVID-19 had fever. As shown in previous studies, although only 43.8% of the patients had fever on admission, 88.7% of all patients had fever during hospitalization [17]. This implies that fever did not present as an initial symptom in all COVID-19 patients; nonetheless, fever occurred as pneumonia symptoms developed. Fever that developed as a result of critical pulmonary infection was frequently observed in patients with severe COVID-19; therefore, temperature monitoring should not be the only screening measure for COVID-19. We suggest that in addition to temperature monitoring, the patient’s contact history and symptoms should be examined to identify individuals who require observation. A higher number of patients showed signs of dyspnea in the severe group than in the non-severe group in our study, indicating that dyspnea was one of the main symptoms among patients with severe COVID-19; thus, a daily evaluation for dyspnea should be conducted among COVID-19 patients. Wang et al. suggest that more frequent chest CT scans should be performed for patients with severe dyspnea to understand the changes and indications in pulmonary imaging [18].

All cases in our COVID-19 study had a decreased lymphocyte count on admission; patients in the severe group had even lower absolute lymphocyte values, as indicated by a median of 0.91 (IQR = 0.59–1.1)×10^9/L. Similar changes in the lymphocyte counts were observed in patients with SARS [19,20]. A previous study regarding lymphocyte subsets indicated that COVID-19 patients had reduced count of total lymphocytes, CD4^+^ T cells, CD8^+^ T cells, B cells, and natural killer cells. Furthermore, severe COVID-19 cases had lower counts than non-severe cases [21]. A study by Qin et al. suggests that COVID-19 might damage lymphocytes, especially T lymphocytes; consequently, the immune system is impaired during the course of the disease [22].The coefficients from our multivariate regression analysis indicated that patients with an absolute lymphocyte value of ≤1.02×10^9/L have an increased risk of experiencing severe COVID-19 (OR = 6.1). This implies that a decrease in the lymphocyte count should be a key predictor in the early diagnosis of severe COVID-19. Research on SARS has shown similar results; a decrease in the lymphocyte count could be an early warning of severe disease [23,24].

This study indicates that patients with elevated concentrations of serum CRP on admission are at an increased risk of experiencing severe COVID-19. The risk of developing severe COVID-19 in patients with a serum CRP of ≥65.08 mg/L is 8.9 times than in patients with a serum CRP≤65.08 mg/L. At elevated concentrations, CRP, which is an acute-phase protein, is correlated with an increased risk of organ failure and death for patients admitted to the ICU. Further, prolonged periods of high CRP concentrations are associated with adverse outcomes [25]. Previous research in Wuhan indicated that increased levels of CRP were indicative of a sustained inflammatory response subsequent to infection with SARS-CoV-2. Additionally, patients with severe COVID-19 had more prominent inflammation [26]. Similar trends of elevated serum CRP were also found in SARS patients [27]; a previous study shows that a CRP concentration of > 47.5mg/L is correlated with an increased risk of death for SARS patients (OR = 5.8) [28].

Our study has several limitations. First, due to the limited ability to collect data and the relatively low morbidity and mortality of COVID 19, the sample size in this study is small, which resulted in relatively few variables being included in the multivariate analysis; it is possible that some significant variables may have been neglected. Second, as this study is a retrospective study, the data collected from the electronic medical records are limited; thus, data about additional significant factors may have not been available. For example, we were unable to analyze the possible causes of higher procalcitonin levels because we lacked data on bacterial infections.

## Conclusions

In this study, we summarized the clinical features of COVID-19 patients and identified the early warning signs of severe COVID-19, which will help physicians determine which patients require further observation. Although this study has some limitations, including a small sample size, few variables included in the multivariate analysis, a retrospective cohort design, and limited data collected from medical records, the results of our study indicate that older age, a decreased lymphocyte count on admission, and an increased concentration of serum CRP could serve as early warning signs in patients who are at risk of developing severe COVID-19. Consequently, we suggest that patients with these clinical characteristics be monitored closely. Further studies should be conducted to confirm the results of our study.
